# Outlier analysis of functional genomic profiles enriches for oncology targets and enables precision medicine

**DOI:** 10.1186/s12864-016-2807-y

**Published:** 2016-06-13

**Authors:** Zhou Zhu, Nathan T. Ihle, Paul A. Rejto, Patrick P. Zarrinkar

**Affiliations:** Oncology Research Unit, Pfizer Worldwide Research & Development, La Jolla Laboratories, 10777 Science Center Drive, San Diego, CA 92121 USA

**Keywords:** Outlier analysis, Functional genomics, Oncology, Cancer, Target identification, Precision medicine, Oncogene addiction, Synthetic lethality

## Abstract

**Background:**

Genome-scale functional genomic screens across large cell line panels provide a rich resource for discovering tumor vulnerabilities that can lead to the next generation of targeted therapies. Their data analysis typically has focused on identifying genes whose knockdown enhances response in various pre-defined genetic contexts, which are limited by biological complexities as well as the incompleteness of our knowledge. We thus introduce a complementary data mining strategy to identify genes with exceptional sensitivity in subsets, or outlier groups, of cell lines, allowing an unbiased analysis without any *a priori* assumption about the underlying biology of dependency.

**Results:**

Genes with outlier features are strongly and specifically enriched with those known to be associated with cancer and relevant biological processes, despite no *a priori* knowledge being used to drive the analysis. Identification of exceptional responders (outliers) may not lead only to new candidates for therapeutic intervention, but also tumor indications and response biomarkers for companion precision medicine strategies. Several tumor suppressors have an outlier sensitivity pattern, supporting and generalizing the notion that tumor suppressors can play context-dependent oncogenic roles.

**Conclusions:**

The novel application of outlier analysis described here demonstrates a systematic and data-driven analytical strategy to decipher large-scale functional genomic data for oncology target and precision medicine discoveries.

**Electronic supplementary material:**

The online version of this article (doi:10.1186/s12864-016-2807-y) contains supplementary material, which is available to authorized users.

## Background

A major challenge in oncology drug discovery is the identification of tumor vulnerabilities that can lead to novel therapeutic targets, and linking these vulnerabilities to specific patient populations that are likely to benefit from pharmacological inhibition of these targets. While historically drug targets have originated from in-depth dissection of cancer biology, more recently tumor genome sequencing efforts such as The Cancer Genome Atlas (TCGA) (http://cancergenome.nih.gov) and International Cancer Genome Consortium (ICGC) (https://icgc.org) have defined the genomic landscape and complexity for an ever growing number of tumor types and subtypes. However, with these approaches, it is becoming increasingly difficult to identify novel oncogenic drivers that are both pharmacologically accessible and applicable to a substantial number of patients.

Functional genomics offers an alternative means for target identification that is complementary to in-depth biology and sequencing. Gene silencing through sequence-specific targeting of mRNAs by RNA interference (RNAi) takes advantage of an endogenous cellular pathway [1, 2] and has become a powerful research tool by enabling high-throughput and systematic loss of function screens in cultured cells and model organisms [3]. One of the largest screens to date is Project Achilles at the Broad Institute, a pioneering effort that has utilized a lentivirally delivered short hairpin RNA (shRNA) library to catalog the dependency of 216 cancer cell lines on 11,000 genes [4].

The analysis of genome-scale RNAi screens typically has focused on *a priori* partition of cell lines based on known biological or genetic contexts, such as the mutation of an established oncogene or tumor suppressor, followed by a comparison of the sensitivity patterns of the two groups to identify genes that, when knocked down, confer preferential sensitivity in one group over the other. This analytical approach has led, for example, to the discovery of *ARID1B* and *SMARCA2* as specific vulnerabilities for *ARID1A* and *SMARCA4*-mutant cancers, respectively [5, 6]. However, the need to pre-define the groups for interrogation represents an inherent limitation due to the incompleteness of our knowledge (e.g. granularity in functional consequence of genomic lesions) as well as biological complexities (e.g. the role of molecular and genetic contexts). Predefining groups, furthermore, calls for a separate analysis for each biological or genetic context, making it impractical to query all contexts of potential interest.

To address these limitations, we have developed a complementary data mining strategy based on patterns of sensitivity in functional genomics screens that requires no *a priori* assumptions about the underlying biology of dependency. Oncogene addiction or synthetic lethality usually results in exceptional response in a subset of tumors or cell lines that are exquisitely vulnerable to knockdown or inhibition of the gene being interrogated [7]. The responder subsets are, by definition, outliers relative to the rest of the population or cell line panel. Taking advantage of this observation, our strategy adapts and extends outlier analysis methodologies to identify genes with a subset of exceptional responders among the screened cell lines. Such a data-driven approach in principle makes it possible to identify vulnerabilities in any biological or genetic context in a single analysis, and also allows for the discovery of novel or complex contexts in which inhibition of specific genes represents a vulnerability that would not have been considered in a pre-defined class comparison analysis.

Outlier analysis has been widely applied to gene expression data for the discovery of cancer-associated genes [8]. It was first described in the identification of a gene fusion in prostate cancer involving two transcription factors, *ERG* and *ETV1* [9], which led to the Cancer Outlier Profile Analysis (COPA) method [9, 10]. Many technically more sophisticated approaches have followed, including model-based pattern recognition for deviation from uni-modality [11–14] and numerical detection for marked high expression in a subset of tumors that is distant from the majority [15–19]. Outlier detection has also been useful in finding drugs with rare but exceptional response in clinical trials [7]. While highly informative, exceptional responder studies in the clinic are constrained by the relatively modest number of biological mechanisms currently targeted by drugs as well as the challenge of following up hypotheses in patients. Large-scale functional genomic studies relieve these restrictions and enable investigating thousands of genes in parallel.

Here we apply an outlier analysis based strategy to functional genomic profiles for systematic oncology target discovery. The utility of such approach is illustrated by the observation that genes with outlier patterns are strongly and specifically enriched with those known to be associated with cancer and relevant biological processes, despite no molecular profiling or any other information being used to drive the analysis. We show that it may enable the identification of novel candidate therapeutic targets, and that the characteristics of the exceptional responder lines could further point to tumor indications and biomarkers of response to guide precision medicine strategies.

## Results

### Identification of genes with outlier sensitivity patterns

To identify genes with an exceptional responder pattern, we used the union of the results output by three diverse methods. They each focus on different features (bimodality, variability, gap) to detect outliers and therefore are considered complementary. Application of these approaches is not intended as a comprehensive comparison of various outlier methodologies; rather we reasoned that together they would provide a more complete set of outliers and outlier genes than any single algorithm. The first two methods were originally developed for outlier analysis of gene expression data: Profile Analysis using Clustering and Kurtosis (PACK) [13], and Outlier Sum (OS) [15]. PACK is a model-based pattern recognition algorithm for discovering bimodal distribution, which first determines the number of clusters in the dataset for each gene and then computes a measure of how much the distribution differs from Gaussianity (kurtosis) for those gene profiles with two clusters. Positive kurtosis indicates clusters of unequal relative size, while negative kurtosis indicates clusters of approximately equal representation. The OS algorithm uses the “outlier-sum” statistic, which is defined using values outside a variability-based numerical limit. It was recently assessed to have the best performance among six closely related outlier techniques [20]. For the third method, inspired by the Q (gap-to-range ratio) statistic utilized in Dixon’s test for outlier detection [21], we devised an intuitive nonparametric approach (details described in Methods; Additional file 1: Figure S1 and Additional file 2)  to explicitly identify genes with dependency patterns where groups of sensitive cell lines are separated by a major “gap” from the bulk population (Gap Analysis Procedure, or ‘GAP’ in short).

**Fig. 1:**
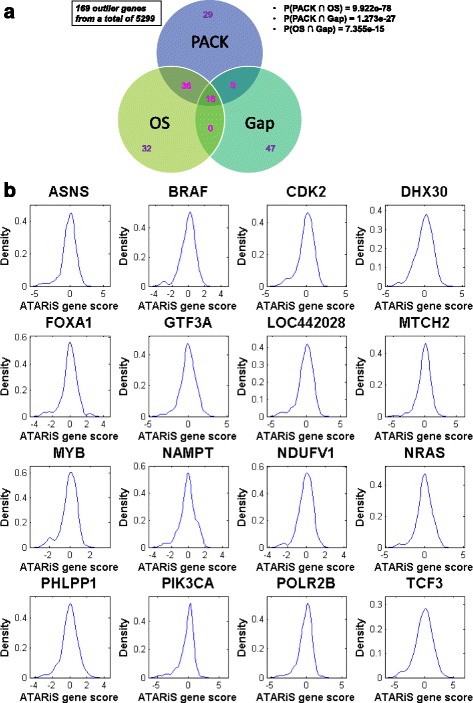
Outlier genes identified with Profiling Analysis using Clustering & Kurtosis (PACK), Outlier Sum (OS) and Gap Analysis Procedure (GAP) methodologies. **a** A summary Venn diagram including statistical significance of pairwise overlap (determined using cumulative hypergeometric probability distribution), with detailed gene list and relevant results included in Additional file 4: Table S2. **b** The ATARiS gene level score distribution for the 16 genes identified by all three outlier methods. A probability density estimate is computed by Gaussian kernel smoothing

All three methods were applied to the Achilles (v2.4.3) ATARiS gene level scores across a diverse panel of 216 cell lines derived from consensus clustering of individual hairpin patterns for on-target effects [22]. The Achilles dataset includes 5299 genes that yielded similarity solution(s) and thereby consensus scores [4]. The PACK algorithm identified 793 genes with bimodal distribution, of which 571 (72 %) had positive kurtosis with one of the two clusters representing a small “outlier” subgroup. As the outlier subgroup could have higher or lower shRNA score than the rest of the panel, we focused on the 105 genes for which the outlier group is more vulnerable to knockdown. The OS and GAP methods led to the identification of genes with a non-random outlier pattern (FDR ≤ 0.05) where statistical significance was estimated using permutation. We also required that the outlier group consist of at least five cell lines to avoid spurious one-off observations, yielding 90 genes from the PACK algorithm, 84 from the OS method, and 72 from the GAP approach. In all, there were 169 unique outlier genes, including 16 that emerged from all three methods (Fig. 1a and b).

### Enrichment for curated cancer genes and oncogenic pathways

**Fig. 2:**
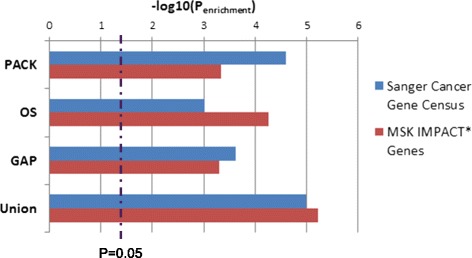
Assessment of genes with significant outlier pattern through comparison with two well-known cancer gene collections. The Cancer Gene Census (CGC) from the Sanger Institute catalogues genes for which mutations have been causally implicated in cancer; The MSK-Integrated Mutation Profiling of Actionable Cancer Targets (IMPACT™) is a curated panel of key cancer genes used for diagnostic genomic testing. “Union” corresponds to 169 combined genes resulting from the three outlier methods (Fig. 1a)

We evaluated the biological relevance of the identified outlier genes by comparing them to two well-known cancer gene collections. The Cancer Gene Census (CGC) from the Sanger Institute catalogues genes for which mutations have been causally implicated in cancer [23] while the MSK-IMPACT™ (Integrated Mutation Profiling of Actionable Cancer Targets) panel is a curated collection of key cancer genes used for diagnostic genomic testing with next-generation sequencing technology (https://www.mskcc.org/msk-impact). Although determined independent of any genetic or other molecular profiling information, the genes with outlier patterns are significantly over-represented by those genetically linked to cancer from both CGC and IMPACT (Fig. 2). Each of the three outlier analysis approaches enriched for established cancer genes, and their union had greater statistical significance than any of them alone (Fig. 2), supporting the complementarity of these methodologies.

We further assessed the validity of our outlier analysis strategy through unbiased signature comparison. Among 1330 gene sets from the Broad Institute’s MSigDB canonical pathway (CP) library (http://www.broadinstitute.org/gsea/msigdb), which collects canonical representations of biological processes compiled by domain experts from pathway databases, the outlier genes are strongly and specifically enriched in those associated with various tumor types including the prominent oncogenes and therapeutic targets *BRAF*, *NRAS*, *KRAS*, *PIK3CA*, *CDK4* and *CTNNB1*, as well as important cellular pathways and processes involved in cancer such as the Wnt, PI3K-mTOR, p53 and Rb-E2F pathways, cell cycle and apoptosis (Table 1). *CDK2* (Fig. 1b), like the targets of the recently approved breast cancer drug Ibrance, is a member of the cyclin-dependent kinase family of Ser/Thr protein kinases whose de-regulation occurs frequently in certain types of cancer [24]. In addition to established cancer genes, our outlier analysis also reveals potentially novel therapeutic opportunities. For instance, asparagine synthetase (*ASNS*) (Fig. 1b), an enzyme that catalyzes the conversion of aspartate and glutamine to asparagine and glutamate in an ATP-dependent manner, has been shown to increase the chemotherapy sensitivity of leukemia cells resistant to L-asparaginase when inhibited [25, 26]. Collectively these results demonstrate that outlier analysis can serve as a useful strategy to identify cancer driver genes from RNAi sensitivity patterns.

**Table 1:** Enriched signatures from the Broad canonical pathway library among outlier genes

Canonical pathway signature	# of outlier genes in the signature	P(enrichment)	FDR
KEGG_PATHWAYS_IN_CANCER	17	1.53E-07	1.63E-04
KEGG_PROSTATE_CANCER	10	2.46E-07	1.63E-04
KEGG_THYROID_CANCER	6	5.01E-06	2.22E-03
PID_WNT_CANONICAL_PATHWAY	4	1.05E-05	3.25E-03
KEGG_ENDOMETRIAL_CANCER	7	1.22E-05	3.25E-03
PID_BETACATENIN_DEG_PATHWAY	4	3.65E-05	7.14E-03
KEGG_MELANOMA	7	3.76E-05	7.14E-03
KEGG_GLIOMA	7	4.44E-05	7.38E-03
REACTOME_CELL_CYCLE_MITOTIC	12	5.20E-05	7.68E-03
KEGG_BLADDER_CANCER	5	1.10E-04	1.47E-02
KEGG_NON_SMALL_CELL_LUNG_CANCER	6	1.56E-04	1.88E-02
PID_HES_HEYPATHWAY	5	1.76E-04	1.88E-02
KEGG_COLORECTAL_CANCER	6	1.84E-04	1.88E-02
REACTOME_SIGNALING_BY_FGFR	7	2.12E-04	2.02E-02
KEGG_ACUTE_MYELOID_LEUKEMIA	6	2.90E-04	2.41E-02
PID_SMAD2_3NUCLEARPATHWAY	6	3.35E-04	2.47E-02
ST_WNT_BETA_CATENIN_PATHWAY	4	3.53E-04	2.47E-02
BIOCARTA_GSK3_PATHWAY	4	3.53E-04	2.47E-02
REACTOME_MITOTIC_G2_G2_M_PHASES	5	3.90E-04	2.60E-02
REACTOME_SIGNALING_BY_FGFR_IN_DISEASE	7	4.24E-04	2.68E-02
PID_MTOR_4PATHWAY	6	5.70E-04	3.29E-02
BIOCARTA_G1_PATHWAY	4	5.94E-04	3.29E-02
SA_G1_AND_S_PHASES	3	6.95E-04	3.29E-02
REACTOME_G0_AND_EARLY_G1	3	6.95E-04	3.29E-02
PID_TCRRASPATHWAY	3	6.95E-04	3.29E-02
PID_RB_1PATHWAY	6	7.27E-04	3.29E-02
PID_IL4_2PATHWAY	5	7.61E-04	3.29E-02
REACTOME_NGF_SIGNALLING_VIA_TRKA_FROM_THE_PLASMA_MEMBRANE	7	7.77E-04	3.29E-02
KEGG_RENAL_CELL_CARCINOMA	6	8.17E-04	3.29E-02
REACTOME_DOWNSTREAM_SIGNALING_OF_ACTIVATED_FGFR	6	8.17E-04	3.29E-02
REACTOME_CELL_CYCLE	12	9.15E-04	3.54E-02
REACTOME_SIGNALING_BY_ERBB2	6	1.27E-03	4.21E-02
KEGG_CHRONIC_MYELOID_LEUKEMIA	6	1.27E-03	4.21E-02
BIOCARTA_CELLCYCLE_PATHWAY	3	1.44E-03	4.55E-02
PID_FOXM1PATHWAY	4	1.66E-03	4.96E-02
REACTOME_SIGNALING_BY_PDGF	6	1.89E-03	4.96E-02
REACTOME_PROLONGED_ERK_ACTIVATION_EVENTS	3	1.94E-03	4.96E-02
PID_E2F_PATHWAY	5	1.97E-03	4.96E-02
PID_ER_NONGENOMIC_PATHWAY	4	1.98E-03	4.96E-02
REACTOME_SIGNALING_BY_EGFR_IN_CANCER	6	2.07E-03	5.10E-02
BIOCARTA_P53_PATHWAY	3	2.55E-03	5.84E-02
KEGG_PANCREATIC_CANCER	5	2.77E-03	6.14E-02
PID_PI3KPLCTRKPATHWAY	4	3.64E-03	7.44E-02
PID_AR_TF_PATHWAY	4	3.64E-03	7.44E-02
BIOCARTA_IGF1R_PATHWAY	3	4.07E-03	8.07E-02
REACTOME_SIGNALING_BY_CONSTITUTIVELY_ACTIVE_EGFR	3	4.07E-03	8.07E-02
KEGG_ERBB_SIGNALING_PATHWAY	5	4.19E-03	8.07E-02
REACTOME_SIGNALING_BY_SCF_KIT	5	4.61E-03	8.63E-02
REACTOME_SIGNALING_BY_ERBB4	5	4.61E-03	8.63E-02
REACTOME_UNFOLDED_PROTEIN_RESPONSE	4	4.74E-03	8.76E-02
BIOCARTA_WNT_PATHWAY	3	6.04E-03	9.36E-02
BIOCARTA_BAD_PATHWAY	3	6.04E-03	9.36E-02
BIOCARTA_IGF1MTOR_PATHWAY	3	6.04E-03	9.36E-02
PID_AURORA_A_PATHWAY	3	6.04E-03	9.36E-02
WNT_SIGNALING	4	6.05E-03	9.36E-02
PID_AR_PATHWAY	4	6.79E-03	0.10
REACTOME_SIGNALLING_BY_NGF	7	7.50E-03	0.10

### Enabling tumor indication and predictive biomarker discoveries

With the identification of genes whose knockdown confers exceptional response, we next asked whether the cell lines making up the outlier groups are enriched in a particular tumor type or subtype, or have molecular features which may be linked to susceptibility to target interference. Such insights would not only guide the precision medicine strategy in oncology drug development for selecting patients who are most likely to benefit from targeted therapies, but also help to further prioritize the most biologically compelling and clinically translatable outlier patterns.

The 216 cell lines from the Achilles dataset were mapped to both general tumor types and common histological subtypes (Additional file 3: Table S1). In the case of breast cancer, both clinical marker (ER/PR/HER2 Triple Negative [TNBC], ER Positive [ER+], HER2 positive [HER2+]) and gene expression (Basal A [BaA_subtype], Basal B [BaB_subtype], Her2 [Her2_subtype], Luminal [Lum_subtype]) based classifications [27] were incorporated. Significant tumor type associations (Additional file 4: Table S2) were found for more than half (86/169) of the genes with outlier patterns. These include well recognized relationships such as *KRAS* in pancreatic and colorectal cancers (*P* = 1.969e-10 and 2.180e-2 respectively), *CDK4* in luminal/ER+ breast cancer (*P* = 3.968e-7 and 9.044e-4 respectively), *BRAF* in skin cancer (*P* = 3.109e-3), *PIK3CA* in breast and gastric cancers (*P* = 1.343e-4 and 1.689e-3 respectively) and *APC* in colorectal cancer (*P* = 2.581e-4), as well as mechanistically supported relationships like *CDK2* in ovarian cancer (*P* = 6.149e-03; Table 2) where *CCNE1*, the cyclin that interacts with *CDK2*, is frequently amplified [28, 29].

**Table 2:** Outlier genes whose predictive genetic biomarkers are the gene itself (in *italic*) or from the same gene family/protein complex, suggesting potential oncogenic addiction or a synthetic lethal relationship. Genetic biomarkers and tumor types are listed by decreasing statistical significance of association

Gene	Genetic biomarker (related)	Tumor type
*APC*	*APC*	*CRC*
ARID1B	ARID1A	
ATP5A1	ATP5H	
*BRAF*	*BRAF*	*CRC;Melanoma*
CDK2	CCNE1;SKP2	Ovarian;Ovarian_serous_adenocarcinoma;Breast Lum_subtype
CRNN	DST;SDF4	ALL;Leukemia/Lymphoma
CTNNB1	APC;PXN;CREBBP	CRC
DHX30	PARP1;DHX40	Breast;Breast HER2+;Breast Her2_subtype
E2F1	RB1	
FOXA1	FOXP4	Breast;Breast Lum_subtype;Breast HER2+;Prostate;
		Breast BaA_subtype;Prostate_adenocarcinoma;
		Breast Her2_subtype;Breast ER+
HNRNPA1	RPL22	
*KRAS*	*KRAS*	*Pancreatic;CRC*
LRPPRC	NFKBIB	Breast Lum_subtype;Breast;CRC; Ovarian_clear_cell_adenocarcinoma;Breast HER2+
*NRAS*	*NRAS*	
*PIK3CA*	*PIK3CA*	*Breast;Gastric;Breast Lum_subtype;Gastric_adenocarcinoma; CRC;Breast HER2+;Breast TNBC;Prostate_adenocarcinoma; Breast Her2_subtype;Breast ER+*
PSMD3	PSMC4;PSMD8	
RBBP4	ACTL6A;GNB4;WDR89	
RBM47	IGF2BP1;MAK16	Breast HER2+;Breast;Breast Her2_subtype; Esophageal_adenocarcinoma
RBMXL1	MAK16;TAF15	
RPS17	RPL38;RPL22	Ovarian_clear_cell_adenocarcinoma;Ovarian
RREB1	ZNF652;GLI2	Breast;Breast HER2+;Breast Her2_subtype
SF3A3	PRPF3	
SLC25A40	SLC2A3;SLCO1B1;SLCO1A2;SLC2A14;LST-3TM12	
SPEN	DHX38;CSTF2T	
TOP2A	PARP1;PPM1D;PRKDC	Breast;Breast Lum_subtype;Breast HER2+
TOPBP1	NBN;BRCA1	
TUBG1	APC	CRC
WDR18	WDR67;GNB2L1;WDR16;NWD1	
*ZMIZ1*	*ZMIZ1*	*Leukemia/Lymphoma;Breast;AML*
ZNF234	ZNF331	Multiple_Myeloma;Ewing_Sarcoma;Leukemia/Lymphoma;Bone
ZNF236	GLI1	

To uncover predictive genetic biomarkers, we focused on the lesions that are most likely to be functional, as tumor genomes are often unstable and thus the vast majority of genetic changes are generally passengers [30]. For somatic mutations, we selected those in hotspot positions as well as nonsense and frameshift events. Hotspots were identified systematically using patient-derived genomic profiles from 20 TCGA tumor types (details described in Materials and Methods), which were subsequently employed to filter cell line mutation data compiled from the COSMIC [31] and CCLE [32] databases. For copy number alterations, we restricted our analysis to high-level amplifications (≥4 copies) and deletions (≤1 copy).

Six (*KRAS*, *NRAS*, *BRAF*, *PIK3CA*, *APC* and *ZMIZ1*) of the 169 genes with outlier patterns are significantly (*P* < 0.05) characterized by their own genetic lesions (Table 2), suggesting oncogenic addiction as the underlying mechanism of their exquisite vulnerability. All of these with the exception of *ZMIZ1* represent prominent oncogenes or tumor suppressors. *ZMIZ1* has previously been identified as a candidate oncogene in multiple murine transposon and insertional mutagenesis screens [33–35].

For 25 additional genes with outlier patterns, the associated predictive genetic biomarkers are either members of the same gene family or components of the same protein complex (Table 2), revealing possible synthetic lethal relationships where lesions in functionally related gene(s) confer special dependency. These include well-documented relationships such as the vulnerability for *CTNNB1* knockdown in the context of *APC* lesions and *E2F1* vulnerability in the context of *RB1* lesions as well as the recently discovered dependency on *ARID1B* in the context of *ARID1A* lesions [5]. In the case of *CDK2*, *CCNE1* amplification is specifically over-represented among its associated exceptional responder cell lines (*P* = 8.215e-04; Table 2), consistent with the tumor type enrichment of ovarian cancer described above. Novel relationships of potential interest were also observed such as *TOP2A* vulnerability with *PARP1* lesions, *HNRNPA1* vulnerability with *RPL22* lesions and *PSMD3* vulnerability for *PSMC4* lesions.

### Distinct dependency and coherence between solid and hematological malignancies

To obtain a global view of tumor cell dependency, we performed unsupervised hierarchical clustering of functional genomic profiles using the genes with outlier dependency patterns (Additional file 4: Table S2). The vast majority of hematological cell lines cluster together by functional data (Additional file 5: Figure S2A and Additional file 6), in contrast to those of solid origin that are more scattered and heterogeneous, despite hematological cell lines representing only 14 % of the total, with tumor types like central nervous system (CNS) and ovarian cancer being equally or more heavily covered by the panel (Additional file 3: Table S1). Even functionally related genes, such as those in the Wnt pathway, differ in points of liability: while cell lines derived from liquid tumors tend to be vulnerable to *TCF3* knockdown, those of solid origin are susceptible to other pathway genes including *GSK3A/B*, *CTNNB1* and *APC* (Additional file 5: Figure S2B).

### Potentially context-dependent oncogenic roles for some tumor suppressor genes

Several outlier genes whose knockdown confers striking vulnerability are tumor suppressors including *APC* [36], *PHLPP1* [37] and *SPEN* [38], while a number of other candidates have been reported to harbor anti-oncogenic activities such as the pro-apoptotic gene *MTCH2* [39] and the anti-metastatic RNA chaperone *RBM47* [40]. Among the five lines most responsive to Adenomatous polyposis coli (*APC*) knockdown, all contain loss of function mutation in the *APC* gene itself and four are of colorectal (CRC) origin (Additional file 7: Figure S3A). Furthermore, they strongly overlap with those dependent on *CTNNB1* (*P* = 1.219e-03; Additional file 7: Figure S3B). Truncating *APC* mutations have been reported to have a dominant negative effect on proliferation, spindle checkpoint control, survival and chromosomal stability [41]. The exceptional responders for *PHLPP1* and *MTCH2* (Fig. 1b) are over-represented by cells from leukemia/lymphoma and colorectal cancer respectively (*P* = 2.211E-8 and 5.178E-3; Additional file 3: Table S2 ). The results therefore support and may further generalize the notion that in certain contexts, tumor suppressor genes can become oncogenic and create specific liabilities.

## Discussion

The analysis of large-scale cell based functional genomics datasets has predominantly focused on identifying vulnerabilities in known biological contexts, such as mutated oncogenes or tumor suppressors. A limitation of this approach is that it requires knowledge of the biology to be interrogated and can generally be described as starting with known biology, then looking for patterns in the data that support it. We have reversed this paradigm and now describe an alternative data mining strategy that starts by looking for profiles indicative of potential dependencies of interest, with no assumptions about the underlying biology of the dependency. It is based on identifying genes with subgroups of exceptionally sensitive cell lines. By definition, these exceptional responders are statistical outliers. We therefore hypothesized that outlier analysis, whose previous application in genomic studies has been limited to gene expression data, would also be useful in investigating RNA interference response patterns. We tested the hypothesis by applying outlier analysis to genome-scale shRNA screen results from Project Achilles at the Broad Institute, and found genes with outlier patterns are significantly and specifically enriched with those that have been causally or genetically linked to cancer as well as related pathways and processes, demonstrating the effectiveness of our novel approach.

As we intentionally selected three diverse methods for outlier analysis, it is not surprising that in addition to common predictions, each algorithm also identifies unique outlier genes (Fig. 1a). The significant over-representation of known cancer genes from each method (Fig. 2) suggests the general utility of the outlier approach is unlikely tied to a specific algorithm, and other outlier analysis methodologies can be similarly employed to decipher functional genomic profiles. The complementarity of the diverse approaches is manifested in the superior enrichment of their output union (Fig. 2), highlighting the heterogeneous pattern of outlier distribution among cancer genes.

The GAP method (Additional file 1: Figure S1) in principle is related to bimodal type of approach in that both detect major separation between outlier and non-outlier groups. This is manifested in the degree of overlap significance between outlier genes identified where Gap shows a stronger agreement with PACK over OS (Fig. 1a). However, unlike bimodal which looks for two normal distributions with distinct means, GAP does not require the outlier group to be Gaussian. Given that the outlier group is often relatively small in size, we believe it is advantageous to circumvent modeling it explicitly in consideration of limited statistical power. The method’s utility is highlighted by some well-known cancer genes such as CDK4, APC and EZH2 are only captured by GAP (Additional file 3: Table S2).

The identification of outliers from functional genomic data also helps to uncover potential indications and predictive biomarkers associated with candidate targets (e.g. ovarian cancer and *CCNE1* amplification for *CDK2* inhibitors) that may guide the development of precision medicine strategies. The National Cancer Institute (NCI) has recently launched the Exceptional Responders Initiative to investigate the molecular factors of tumors associated with exceptional treatment responders of cancer patients to drug therapies [42]. Outlier analysis of functional genomics data from large-scale gene silencing provides an opportunity to address similar questions for thousands of genes in parallel using pre-clinical models.

The set of genes with outlier dependency patterns may also provide a useful framework for a global view of tumor cell dependency. Hematological lines have a unique vulnerability pattern that appears more homogenous than their solid tumor counterparts. This may reflect their evolutionary history where leukemias and lymphomas likely require fewer rounds of clonal expansion [43–45] as their precursor cells are already mobile and invasive [46]. It is also possible that some technical factors (e.g. conditions in cell culturing) may contribute to the observed difference. With experimental validation, the grouping of tumor cells by functional dependency could lead to important insights on the design of more sophisticated molecular biomarker strategies of both positive and negative selections for basket trials.

Our apparently counter-intuitive observation that the knockdown of several tumor suppressor genes resulted in striking vulnerability in a subset of tumor cells suggested they could be oncogenic in specific circumstances. This is analogous to the recent finding in the ARID1 family whereby inactivation of *ARID1A* creates a special dependence on the related tumor suppressor gene *ARID1B* [5] and the “abnormal” (*ARID1A*-less) SWI/SNF complex is pro-oncogenic. The presence of additional tumor suppressors like *ARID1B* and *APC* among outlier genes suggests that these are unlikely to be isolated cases and more may have context-dependent dual properties, presenting a challenge to the simple binary classification of cancer genes as either oncogenes or tumor suppressors, and bringing up the possibility of expanding the druggable genome to include tumor suppressors when coupled with an appropriate precision medicine strategy.

While outlier vulnerability analysis of Achilles RNAi sensitivity pattern has successfully uncovered many well-known cancer genes along with their tumor type and genetic biomarker relationships, it also has a few notable misses. *EGFR* and *ERBB2*, two established oncology therapeutic targets, do not appear to harbor a significant exceptional responder group distinct from the population (Additional file 8: Figure S4). We should note that the outlier approach, as any analytical strategy, is contingent upon the quality of the input: technological limitations such as hairpin off-target seed effects [47–51] have an inevitable negative impact on analysis results. Even though we used ATARiS gene-level solutions which have on-target signals greatly amplified as input, our analysis is still not completely immune from this complexity and novel findings in particular need to be experimentally confirmed for proper interpretation. We have described here the application of outlier analysis to only a single large functional genomics dataset; however, the identification of many known cancer genes and relationships provides confidence and proof-of-principle for the general approach.

Whereas we have used Achilles shRNA patterns as an example, outlier analysis should be equally relevant to datasets from alternative functional genomic technologies including siRNA and CRISPR [52–54], as well as to other shRNA screen data. Since outliers are rare events by definition, their detection requires a sufficiently large population as with genome-wide gene expression datasets. Furthermore, for most of the outlier techniques to work effectively, the overall input data (after normalization) should follow a symmetric and preferentially normal distribution without the confounding of excessive technical outliers as discussed above. The outlier analysis approach is not only useful for mining of gene-level values but also lower-level data such as those from individual hairpins where the consistency between outlier responders could provide another means to select for on-target effects.

## Conclusions

With the rapid evolution of functional genomic technologies, there is an ever growing demand for analytical strategies to maximize discoveries from the large amounts of data being generated. The current analyses typically focus on genes whose knockdown enhances response in pre-defined molecular contexts and thus are inherently limited in their ability to reveal new disease-relevant biology. Here we tackle this important conceptual problem and demonstrate one solution by introducing a novel strategy to identify tumor vulnerabilities from functional genomic profiles based on patterns of responsiveness alone. It takes advantage of the observation that oncogene addiction or synthetic lethality generally manifests itself in the exquisite sensitivity of a subset of tumors or cell lines, and therefore is built upon the identification of genes with outlier drop-out pattern. We thus expand the utility of outlier analysis, whose application in genomics thus far has been restricted to gene expression data, towards the mining of functional genomic profiles.

We show that genes with outlier vulnerability pattern are strongly and specifically enriched with those known to be associated with cancer and relevant biological processes, demonstrating its utility for the identification of therapeutic targets. The characteristics of the outlier lines can further reveal tumor indications and biomarkers of response associated with candidate targets to guide the development of precision medicine strategies. In addition, it provides a useful framework for a global view of tumor cell dependency, which led to the observation of distinct sensitivity and coherence between solid and hematological malignancies. The counter-intuitive finding of several tumor suppressors with outlier sensitivity patterns challenges the simple binary classification of cancer genes as either oncogenes or tumor suppressors, and generalizes the notion that tumor suppressors could play context-dependent oncogenic roles. Therefore, our novel analytical approach described here offers a valuable alternative means to mine fast-growing functional genomic data in an unbiased manner for discoveries that may lead to the next generation of oncology medicines.

## Methods

### Achilles data

Project Achilles is a systematic effort aimed at identifying and cataloging genetic vulnerabilities across hundreds of genomically characterized cancer cell lines [4]. The project uses genome-wide genetic perturbation reagents (shRNAs) to silence or knock down individual genes and identifies those genes that affect cell survival. The latest version (2.4.3) was downloaded from the project data portal (http://www.broadinstitute.org/achilles). The file Achilles_QC_v2.4.3.rnai.Gs.gct containing ATARiS [22] gene level scores for 216 cell lines that pass quality control (*p* ≤ 0.05) [4] was used as input for our outlier analysis below.

### Outlier analysis

1. PACK
  Profile Analysis Using Clustering and Kurtosis (PACK) algorithm implemented in the vabayelMix library of R [13] was applied to Achilles data for outlier identification. We filtered its output for bimodal genes of positive kurtosis (i.e. unequal relative mass), with outlier group more vulnerable to drop-out and containing at least 5 cell lines.
2. Outlier Sum
  The Outlier Sum (OS) algorithm [15] was applied to gene-summarized Achilles data for outlier identification. The outlier-sum statistic for each gene *i* was defined to be “one-sided” as the sum of the values that are smaller than the limit q_25_(i)-IQR(i):
  $$ {\displaystyle \sum }{x}_{ij}\cdot I\left[{x}_{ij}<{q}_{25}(i)-IQR(i)\right] $$
  where inter-quartile range (IQR) = q_75_(i)-q_25_(i), x_ij_is the drop-out score for gene i in cell line j and I represents the conditional IF test of whether x_ij_ is lower than this limit (1 if true and 0 if false). Statistical significance was estimated through 10,000 full permutation of the data matrix with outlier-sum statistic calculated in the same manner. We focused on genes with at least 5 outlier cell lines and the false discovery rate (FDR) was determined using the Benjamini–Hochberg procedure [55].
3. Gap Analysis Procedure (GAP)
  We devised a gap-based measure for outlier identification (Additional file 1: Figure S1). Let *x*_*i*,*j*_ be the drop-out value for gene *i* in cell line *j*, reorder the values for each gene so that
  $$ {y}_{i, 1}\kern0.37em \le {y}_{i, 2}\le \dots .\ \le {y}_{i,m}{\le}_i\tilde{x}\le {y}_{i,m+ 1}\le \dots .\ \le {y}_{i,n} $$
  where ${\tilde{x}}_i$ is the median for gene *i* across cell lines.
  An outlier sum statistics was computed as following based on gaps (G) between adjacent data points:
  $$ {S}_i=\left\{\begin{array}{c}\hfill {\displaystyle \sum_{j=1}^k}{y}_{i,j}\kern0.36em  if\ \underset{1\le j\le m}{ \max }G\left({y}_{i,j}\right)\ge\ \alpha \left({y}_{i,n} - {y}_{i,1}\right)\ \hfill \\ {}\hfill 0\kern2.05em  if\ \underset{1\le j\le m}{ \max }G\left({y}_{i,j}\right) < \alpha \left({y}_{i,n} - {y}_{i,1}\right)\hfill \end{array}\right. $$
  where gap *G*(*y*_*i*,*j*_) = *y*_*i*,*j* + 1_ − *y*_*i*,*j*_ and *k* = max_1 ≤ *j* ≤ *m*_*j* such that *G* (*y*_*i*,*j*_) ≥ *α* (*y*_*i*,*n*_ − *y*_*i*,*1*_) For α, we used an arbitrary value of 0.05. Statistical significance was estimated through 10,000 full permutations of the data matrix with the gap-based outlier sum statistic calculated in the same manner. We focused on genes with at least 5 outlier cell lines and the false discovery rate (FDR) was determined using the Benjamini-Hochberg procedure [55].

### Most likely functional genetic lesions

To avoid cell culture artifacts and technical biases in cell line collection, mutation hotspots were identified in a systematic and unbiased manner from patient-derived somatic mutation profiles. The Cancer Genome Atlas (TCGA) level 3 mutation data (.maf files) for 20 diverse tumor types (BLCA, BRCA, CESC, COAD, GBM, HNSC, KIRC, KIRP, LAML, LGG, LUAD, LUSC, OV, PAAD, PRAD, READ, SKCM, STAD, THCA, UCEC) were downloaded through firehose (http://gdac.broadinstitute.org/). We filtered for non-silent coding mutations (De_novo_Start_InFrame, De_novo_Start_OutOfFrame, Frame_Shift_Del, Frame_Shift_Ins, In_Frame_Del, In_Frame_Ins, Missense, Nonstop, Read-through, Translation_Start_Site) and excluded those mapped to pseudogenes. Nonrandom Mutation Clustering (NMC) algorithm [56] was applied to the resulting “Pan-Cancer” mutation profile, with multiple hypothesis testing corrected by the Benjamini-Hochberg procedure [55]. We focused on significant mutation hotspots (FDR ≤ 0.05) of no more than 50 nucleotides. Such length was selected as a compromise for both activating and inactivating missense mutations as activating ones tend to be rather focal [43] while inactivating ones often span a relatively relaxed region/domain [57].

The non-silent coding mutation profiles for cell lines were obtained using Cell Index database (CELLX) [58] based on genetic data compiled from the Cancer Cell Line Encyclopedia (CCLE) [32], Sanger Catalog of Somatic Mutations in Cancer (COSMIC) [59] and Sanger Wellcome Trust Genomics of Drug Sensitivity in Cancer (GDSC) [60]. To select for most likely functional mutations, we filtered for those located at patient-derived hotspots as identified above, as well as loss-of-function (LOF) ones by mutation type of nonsense and frameshift. Copy number segments for cell lines were also obtained using CELLX. Amplifications and deletions for a gene were defined as copy number segments overlapping the gene of at least four copies and at most one copy respectively, if any.

### Gene pairs related by families and protein complexes

1012 human gene families together with their associated members were downloaded from genenames.org (http://www.genenames.org/), a curated online repository of HGNC-approved gene nomenclature, gene families and associated resources. Protein complex relationships were obtained from the Comprehensive Resource of Mammalian protein complexes (CORUM) database which provides a resource of manually annotated protein complexes from mammalian organisms including function, localization, subunit composition and literature reference [61, 62]. 1846 such complexes are from human sources. For each pair of outlier gene and its predictive genetic biomarker (see *Enrichment analysis* below), we consider them functionally related if they belong to the same human gene family or protein complex based on the annotations described here.

### Clustering analysis

Unsupervised hierarchical clustering was performed with correlation as similarity metric and average linkage as clustering method using Cluster 3.0 software (http://bonsai.hgc.jp/~mdehoon/software/cluster/software.htm). Non-outliers represent non-sensitive hits and are thus “flattened” to zero. To focus on most variable features, 165 out of the 169 genes with significant outlier sensitivity pattern (Additional file 3: Table S2) were used in the clustering analysis as they each have less than a quarter of total cell lines classified as outliers. The clustering results were visualized using TreeView program [63] and heatmap.2 function of ‘gplots’ package in R (http://www.inside-r.org/packages/cran/gplots/docs/heatmap.2).

### Enrichment analysis

The statistical significance for enrichment of outliers in a tumor (sub)type or with genetic biomarker was calculated as follows:

$$ \mathrm{P}=1-{\displaystyle {\sum}_{i=0}^{x-1}\frac{\left(\begin{array}{c}\hfill K\hfill \\ {}\hfill i\hfill \end{array}\right)\left(\begin{array}{c}\hfill M-K\hfill \\ {}\hfill N-i\hfill \end{array}\right)}{\left(\begin{array}{c}\hfill M\hfill \\ {}\hfill N\hfill \end{array}\right)}}, $$

where M = total of cell lines, K = number of outliers (the union from the three methods), N = number of cell lines from the tumor (sub)type or with likely functional genetic lesion, and x = number of outliers in the tumor (sub)type or with genetic biomarker. The probability of obtaining at least the observed number of common outlier genes from a pair of methods by chance was determined in an analogous manner, where M = total number (5299) of genes with ATARiS consensus solution, K and N = number of outlier genes identified by each method respectively, and x = number of overlapping outlier genes identified by both methods.

### Gene signature analysis

Gene signature enrichment analysis was performed by comparing outlier genes with those from the CGC catalogue and MSK-IMPACT™ panel as well as those from the Broad MSigDB’s canonical pathway (CP) library. Statistical significance was determined using cumulative hypergeometric probability distribution as previously described [64], where the total number of genes was based on those with ATARiS gene consensus solution [4, 22] and multiple hypothesis testing was corrected by Benjamini-Hochberg procedure [55].

## Abbreviations

ATARIS, analytic technique for assessment of RNAi by similarity; CCLE, cancer cell line encyclopedia; CGC, cancer gene census; CORUM, comprehensive resource of mammalian protein complexes; COSMIC, catalogue of somatic mutations in cancer; CP, canonical pathway; CRISPR, clustered regularly interspaced short palindromic repeats; FDR, false discovery rate; GAP, gap analysis procedure; GDSC, genomics of drug sensitivity in cancer; ICGC, international cancer genome consortium; IMPACT, integrated mutation profiling of actionable cancer targets; LOF, loss of function; MSigDB, molecular signatures database; NCI, National Cancer Institute; NMC, nonrandom mutation clustering; OS, outlier sum; PACK, profile analysis using clustering and kurtosis; RNAi, RNA interference; shRNA, short hairpin RNA; siRNA, small interfering RNA; TCGA, the cancer genome atlas

## Additional files

Additional file 1: Figure S1. A schematic diagram of the GAP approach. Its matlab implementation is included as Additional file 2. (DOCX 189 kb) Additional file 2: An implementation of the GAP approach in matlab. (PDF 43 kb) Additional file 3: Table S1. Tumor type and common subtype classification of Achilles cell lines. (XLSX 16 kb) Additional file 4: Table S2. The complete list of genes with outlier patterns along with tumor type indication and related predictive biomarker results (sorted by decreasing statistical significance of association), if any. The table indicates for each gene which of the three analysis methods determined it to be associated with an outlier responder pattern. (XLSX 16 kb) Additional file 5: Figure S2. Unsupervised hierarchical clustering of tumor cells by functional dependency. (A) Grouping of drop-out patterns using outlier genes, where rows correspond to genes and columns correspond to cell lines. Non-outlier genes are colored in grey as they represent non-sensitive hits. The right inset provides a zoom-in view of the yellow cluster with the vast majority of hematological lines. The full size heatmap, dendrograms and labels have been included as Additional file 6. (B) Grouping using outlier genes from the Wnt pathway. (DOCX 1164 kb) Additional file 6: The complete clustering diagram shown in Additional file 5: Figure S2A including heatmap, dendrograms and row/column labels in full page size. (JPG 10376 kb) Additional file 7: Figure S3. The outlier pattern for APC. (A) The kernel density plot of ATARiS gene level score for APC and details on the five outlier cell lines most vulnerable to its knockdown. (B) Unsupervised hierarchical clustering of tumor cells by functional dependency on outlier genes from the Wnt pathway, with APC highlighted in yellow. (DOCX 395 kb) Additional file 8: Figure S4. The ATARiS gene level score distribution for (A) EGFR and (B) ERBB2. A probability density estimate is computed by Gaussian kernel smoothing. (DOCX 49 kb)
