# Supplementary material for: Outlier analysis of functional genomic profiles enriches for oncology targets and enables precision medicine
Source: BMC Genomics. 2016 Jun 13;17:455. doi: 10.1186/s12864-016-2807-y (PMC4907009; doi:10.1186/s12864-016-2807-y)
Supplement: Additional file 1: Figure S1. — A schematic diagram of the GAP approach. Its matlab implementation is included as Additional file 2. (DOCX 189 kb) [file 12864_2016_2807_MOESM1_ESM.docx]

sFig 1
