# Supplementary material for: Outlier analysis of functional genomic profiles enriches for oncology targets and enables precision medicine
Source: BMC Genomics. 2016 Jun 13;17:455. doi: 10.1186/s12864-016-2807-y (PMC4907009; doi:10.1186/s12864-016-2807-y)
Supplement: Additional file 2: — An implementation of the GAP approach in matlab. (PDF 43 kb) [file 12864_2016_2807_MOESM2_ESM.pdf]

```
%sFile1:GAP.m
%input#1: Num.txt contains a numerical matrix where rows correspond to
genes/hairpins and columns correspond to cells/samples;
%input#2: Name.txt contains row labels (i.e. gene/hairpin names) of
Num.txt
%output: OutlierSummary.txt

load Num.txt;
fid=fopen('Name.txt','r');
names={};
while ~feof(fid)
    temp={fgetl(fid)};
    names=[names;temp];
end
fclose(fid)

out='OutlierSummary.txt';
outFile=fopen(out,'w');

a=0.05;

for i=1:size(Num,1)
      temp1=Num(i,:);
      temp1(isnan(temp1))=0;
      A=sort(temp1);
      B=min(find(A>=median(temp1)))-1;
      array=[];
      for j=1:B-1
        gap=A(j+1)-A(j);
        array=[array;gap];
      end
      C=max(array);
      thr=range(temp1)*a;
      if C >= thr
            k=max(find(array>=thr));
            S=sum(A(1:k));
            if k >= 5
                  fprintf(outFile,'%s\t%d\t%f\n',names{i},k,S);
            end
      end
end
```
