# Supplementary figures and images for: Outlier analysis of functional genomic profiles enriches for oncology targets and enables precision medicine
Source: BMC Genomics. 2016 Jun 13;17:455. doi: 10.1186/s12864-016-2807-y (PMC4907009; doi:10.1186/s12864-016-2807-y)

sFig 2

(A)

(B)


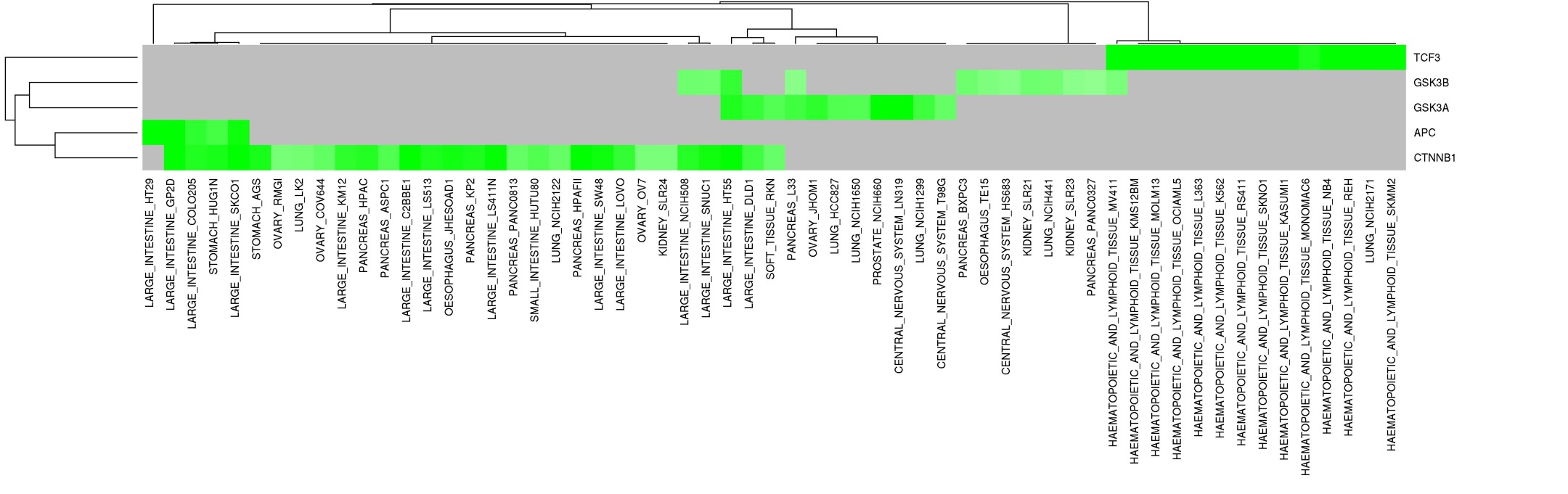

Supplement: Additional file 5: Figure S2. — Unsupervised hierarchical clustering of tumor cells by functional dependency. (A) Grouping of drop-out patterns using outlier genes, where rows correspond to genes and columns correspond to cell lines. Non-outlier genes are colored in grey as they represent non-sensitive hits. The right inset provides a zoom-in view of the yellow cluster with the vast majority of hematological lines. The full size heatmap, dendrograms and labels have been included as Additional file 6. (B) Grouping using outlier genes from the Wnt pathway. (DOCX 1164 kb) [file 12864_2016_2807_MOESM5_ESM.docx]

sFig 4

(A)


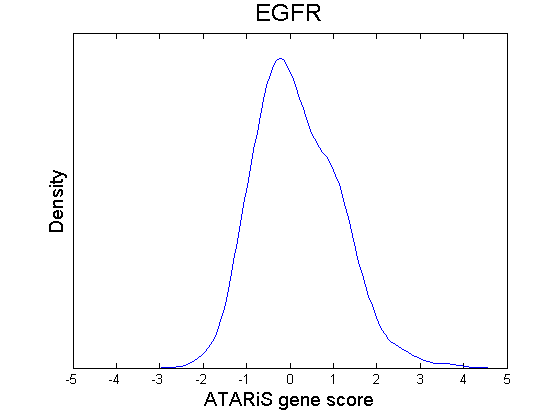


(B)


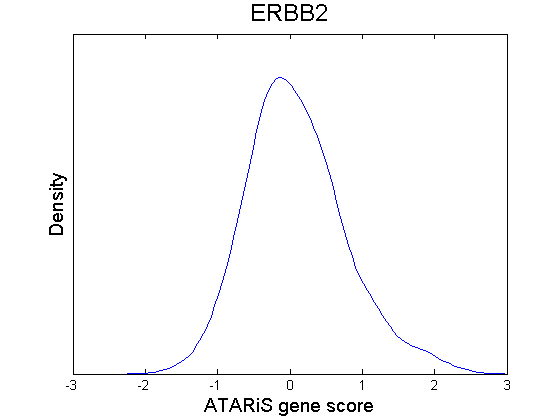

Supplement: Additional file 8: Figure S4. — The ATARiS gene level score distribution for (A) EGFR and (B) ERBB2. A probability density estimate is computed by Gaussian kernel smoothing. (DOCX 49 kb) [file 12864_2016_2807_MOESM8_ESM.docx]
