# Supplementary material for: Outlier analysis of functional genomic profiles enriches for oncology targets and enables precision medicine
Source: BMC Genomics. 2016 Jun 13;17:455. doi: 10.1186/s12864-016-2807-y (PMC4907009; doi:10.1186/s12864-016-2807-y)
Supplement: Additional file 7: Figure S3. — The outlier pattern for APC. (A) The kernel density plot of ATARiS gene level score for APC and details on the five outlier cell lines most vulnerable to its knockdown. (B) Unsupervised hierarchical clustering of tumor cells by functional dependency on outlier genes from the Wnt pathway, with APC highlighted in yellow. (DOCX 395 kb) [file 12864_2016_2807_MOESM7_ESM.docx]

sFig 3

(A)

(B)
